# Reactivity of Dogs' Brain Oscillations to Visual Stimuli Measured with Non-Invasive Electroencephalography

**DOI:** 10.1371/journal.pone.0061818

**Published:** 2013-05-01

**Authors:** Miiamaaria V. Kujala, Heini Törnqvist, Sanni Somppi, Laura Hänninen, Christina M. Krause, Outi Vainio, Jan Kujala

**Affiliations:** 1 Lyon Neuroscience Research Center, INSERM U1028 - CNRS UMR5292, Bron, France; 2 Department of Equine and Small Animal Medicine, Faculty of Veterinary Medicine, University of Helsinki, Helsinki, Finland; 3 Department of Biomedical Engineering and Computational Science, Aalto University, Espoo, Finland; 4 Research Center for Animal Welfare, Faculty of Veterinary Medicine, University of Helsinki, Helsinki, Finland; 5 Cognitive Science, Institute of Behavioural Sciences, Faculty of Behavioural Sciences, University of Helsinki, Helsinki, Finland; 6 Department of Production Animal Medicine, Faculty of Veterinary Medicine, University of Helsinki, Helsinki, Finland; 7 Brain Research Unit, O.V. Lounasmaa Laboratory, Aalto University, Espoo, Finland; University College of London - Institute of Neurology, United Kingdom

## Abstract

Studying cognition of domestic dogs has gone through a renaissance within the last decades. However, although the behavioral studies of dogs are beginning to be common in the field of animal cognition, the neural events underlying cognition remain unknown. Here, we employed a non-invasive electroencephalography, with adhesive electrodes attached to the top of the skin, to measure brain activity of from 8 domestic dogs *(Canis familiaris)* while they stayed still to observe photos of dog and human faces. Spontaneous oscillatory activity of the dogs, peaking in the sensors over the parieto-occipital cortex, was suppressed statistically significantly during visual task compared with resting activity at the frequency of 15–30 Hz. Moreover, a stimulus-induced low-frequency (∼2–6 Hz) suppression locked to the stimulus onset was evident at the frontal sensors, possibly reflecting a motor rhythm guiding the exploratory eye movements. The results suggest task-related reactivity of the macroscopic oscillatory activity in the dog brain. To our knowledge, the study is the first to reveal non-invasively measured reactivity of brain electrophysiological oscillations in healthy dogs, and it has been based purely on positive operant conditional training, without the need for movement restriction or medication.

## Introduction

Recently, the interest in studying cognition of domestic dogs *(Canis familiaris)* has increased tremendously. Although the species is phylogenetically further away from humans than non-human primates, its evolution has been affected by a long domestication period and, more lately, a selection of behavioral traits by humans via breeding of the dog species. Accordingly, behavioral studies of dog cognition have revealed similarities of dog behavioral traits to humans [1]–[3]. Dogs have been found to engage in gaze following [4] similarly to human babies [5], exhibit selective imitation [6] similarly to human infants [7], to observe photos of faces [8] roughly similarly to human adults [9] and to link photos of objects to objects themselves [10].

Some features of dog behavior thus suggest similarities in cognitive processing of humans and dogs. However, not much is yet known about the underlying neural processes of dogs during perception and cognition, or the possible similarities to neural processes of humans. Brain function of dogs has been studied in the past mainly by recording activity with electroencephalography (EEG) directly from the brain, by sedating the animals and restraining their movements, and by putting them down after the experiment. Most of the functional brain research of dogs has explored epilepsy [e.g., [11], although some studies have described features of the nervous system functionality, such as oscillatory EEG activity during sleep [12] or awake state [13], or visual evoked potentials to flashes of light [14]–[16]. However the intracranial measurements, with the need to restrain and medicate the animals, do not readily allow the study of the nervous system function during cognitive events. Hence much of the underlying neural functionality of dog cognition remains unresolved.

In humans, the basic functionality of the brain oscillatory activity is well characterized in neurophysiological experiments; for example, the suppression of alpha frequency range brain oscillations in humans due to opening the eyes (respective to closing the eyes) has been known since Berger [17]. More recently, the electrophysiological oscillatory activity and its correlates to cognition have been studied both with intracranial EEG measured directly from the brain of epileptic patients as well as with non-invasive neurophysiological measurements from outside the scalp (for reviews, see e.g., [18]–[22]).

In the current study, we utilized a completely non-invasive EEG measurement in a group-level study on dogs. To address the basic oscillatory functionality of the visual processing within a dog brain, eight purpose-bred beagle dogs were taught, with positive operant conditional training, to lay still and observe visual stimuli presented on a computer screen in front of them, while EEG was recorded non-invasively. The aim of the study was to characterize the group-level basic oscillatory activity in domestic dogs applying a non-invasive method. In principle, the research setting was comparable to standard human visual experiments where the subjects observe the stimuli while their brain activity is measured.

## Materials and Methods

### Ethics statement

The study was performed in strict accordance with the The Finnish Act on Animal Experimentation (62/2006), with the European convention for the protection of vertebrate animals used for experimental and other scientific purposes (Directive 86/609/EEC) fully implemented. All the experimental procedures of the study were approved by the Ethics Committee of the University of Helsinki (approval #STH367A/ESLH-2008-04236/Ym-23). No invasive procedures were applied, and only positive reinforcement was used in the animal training. During the measurements, the dogs were fully alert and conscious at all times with no medication, and neither mechanical nor manual restraint was applied.

### Subjects

Subjects were eight (8) clinically healthy, neutered purpose-bred beagles from five different litters. The dogs were raised as a social group and housed in a group kennel [6 males, 2 females, weighing 12.9. ± 1.9 kg (mean ± SD)], and all dogs were 4 years old at the time of the measurements. Purpose-bred dogs formed the subject group, since the aim was to establish a “baseline” for studies on dog visual perception with animals who have very similar backgrounds, to avoid excess variation due to environmental effects. Furthermore, the subject dogs of the same breed, with comparable head sizes and forms, enabled the comparison of the responses at a group-level.

### Stimuli

Stimuli were color photos of upright and inverted human and dog faces, obtained from internet photo databases (www.123rf.com and www.bigstockphoto.com) and from personal collections. Face images were used due to their ecological valence for the dogs: in our previous experiment, dogs were found to gaze face stimuli more than other stimulus categories (such as toys or letters, [8]). Furthermore, the face images were used due to the concurrent eye tracking experiment with a different agenda. All the faces were detached from their photographic background and placed in the middle of a medium grey background. In total, the stimuli comprised 36 upright photos of human faces and 39 upright photos of dog faces, and 3 inverted photos of human and 3 of dog faces. Each picture was repeated 2–7 times resulting in a total of 240 image presentations.

### Stimulus presentation procedure

The photos were displayed on a standard 22″ LCD monitor, overlaid on a gray background screen of 1680 × 1050 pixels and presented with a frame rate of 60 Hz. The stimulus objects were positioned on the center of the screen and covered 13.8 ± 1.3% (mean ± SD) of the total screen size, resulting in approximately 14.6 × 16.0 cm (width × height) size on the grey background of 47.4 × 29.7 cm in size. Stimulus presentation was controlled with Presentation® software (http://nbs.neuro-bs.com/) run on a PC.

The stimuli were presented, in a pseudorandomized order, at a distance of 70 cm, while the dogs laid still on a 10 cm thick Styrofoam mattress and leaned their jaw on a purpose-designed u-shaped chin rest. Each stimulus was shown for 1.5 s with an inter-stimulus-interval of 500 ms, within 6 separate stimulus blocks of 8–12 stimuli per block, and 2 min 11 s ± 10 s (mean ± SEM) rewarding periods between blocks. During the rewarding periods, the dog was rewarded with a piece of food and let to settle again on the measurement mattress. At the end of the measurement session of 6 stimulus blocks, the dogs continued to lay still in front of the monitor for 1–5 periods of 10–40 s, with food rewards in between these periods, to record “resting” data with no stimuli. During the resting periods, the dogs' eyes were open and they continued to gaze forward at the blank stimulus screen with a cardboard wall behind the screen, but no additional visual input was given.

The total measuring time was about 20 minutes per session (range 12–39 min); only one session was recorded per day per dog. The data were gathered in four recording sessions, each during separate day. The dogs' eye movements were recorded simultaneously with a iView X™ RED (SensoMotoric Instruments GmbH, Germany) and used to confirm the dogs' attention to the stimuli; the eye gaze data itself is a part of another study.

### Training and EEG measurement

**Figure 1:**
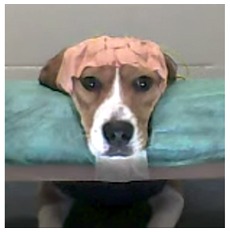
Experimental setup. Capture of the webcam, showing a dog resting its head to the chin rest and observing the stimulus screen during the non-invasive measurement, with electrodes attached to the top of the skin in a comparable fashion to standard human EEG measurements.

During the preceding 1.5 years to the study, the dogs were trained about twice a week to come to the measurement room; to wear the Unilect™ neonatal EEG electrodes (type 40555 with bio-adhesive solid gel, 22 × 22 mm) designed for newborn babies (Unomedical a/s, Denmark) and a dog vest carrying the portable EEG amplifier (weighing 200 g); to settle in the measurement mattress without being commanded; to rest their head at a customarily-built chin rest while the experimenter was positioned behind an opaque barrier; and to stay still in front of the computer screen (see Figure 1). Dogs were trained using an operant-positive conditioning method (clicker). The dogs were not restrained and they could move if so wished; however they were positively reinforced to stay still during the task.

**Figure 2:**
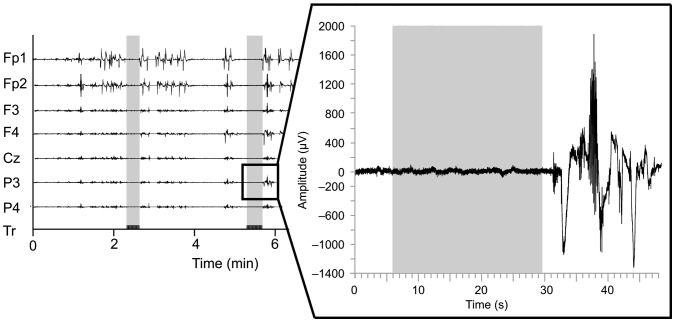
An example of the raw data from all the channels. The stimulus triggers (Tr) are shown at the bottom channel as a series of square waves and the respective time points are shaded in vertical gray sections through all the EEG channels (Fp1–P4). The magnification of data on the right illustrates the quality of the data; the dog has stayed still during the stimuli and moved only after the stimulus block to receive its reward.

To attach the electrodes to the skin, the hairs from the top of the dog's head was shaved and the skin was rubbed with NuPrep™ gel and cleaned with isopropyl alcohol to ensure a sufficient contact of the electrodes to the skin. Subsequently, drops of instant adhesive (cyanoacrylate) was applied to the edges of the electrode pads, and a medical skin tape was applied on top of the electrodes to ensure their attachment. The EEG data were acquired with an ambulatory Embla® Titanium™ -recorder and RemLogic™ 2.0 –software (Embla Systems, Colorado, USA). The EEG setup comprised 7 electrodes on the top of the skin, an electrode in each ear (y-linked for a reference), and a ground electrode in the lower back. The impedances of the electrodes were measured before the experiment, between the stimulus blocks and after the experiment. The EEG signals were band-pass filtered to 0.15–220 Hz and digitized at 512 Hz. Figure 2 shows an example of the raw data. As is evident from the traces, during the TASK blocks the dogs were fully engaged in the task and remaining still, yielding stable EEG data; the selected time-intervals of the EO-REST condition showed similar EEG data quality.

### Data analysis

#### Spontaneous rhythmic activity (TASK vs. EO-REST)

To characterize the spontaneous rhythmic activity, the oscillatory brain rhythms were categorized as two conditions: visual task-related activity (TASK) and as eyes open but resting activity (EO-REST). The TASK activity was recorded during the stimulus blocks, from the onset of the first trigger of the first stimulus to 1 s after the onset of the last trigger of each stimulus block, including ISIs (average duration of a stimulus block 19 sec ± 3 sec, mean ± SD). The EO-REST activity was recorded during the resting periods at the end of the measurement (average duration of a rest block 26 sec ± 14 sec), during which the dog was lying still with eyes open and gazing a blank screen. The power spectra, during both the TASK and EO-REST periods, were calculated using Welch's averaged, modified periodogram method [23]. In the calculation, each TASK and EO-REST period was divided into partially (50%) overlapping 512 sample long segments. Segments that contained external artifacts or inadequate impedance (time intervals with activity over 200 µV in any EEG channel indicating muscle activity and eye movement -related artefacts) were excluded from further analysis. To further reduce the effects of muscle artefacts on the data, independent component analysis (ICA) was applied [24], separately for each TASK and EO-REST block. For each block, the most prominent artefact component was identified based on the inspection of the topography and spectral content of the components, and its influence was removed. Thereafter, the ICA-processed data segments were windowed using a Hanning window and detrended. Subsequently, the Fast Fourier Transform (FFT) was applied, and the obtained FFTs were averaged across segments.

To compare the spontaneous oscillatory rhythms during TASK and EO-REST at a group level, data of individual dogs were first normalized with the mean power levels of the TASK condition across all frequencies and EEG sensors. Thereafter, the peak frequency in each sensor was determined from the power spectra averaged across dogs and conditions. The possible difference between the two conditions was then tested in frequency bands of ± 3 Hz around these peak frequencies using a paired-samples t-test.

#### Induced oscillatory activity (STIMULUS vs. BASELINE)

To identify and characterize the induced oscillatory activity (induced by and time-locked to the visual stimulus, but not necessarily phase-locked), we utilized an approach commonly used in the analysis of human oscillatory brain electrophysiological activity called time-frequency representation (TFR) [25], [26]. The TFR displays the frequency content of the signal as a function of time, thus enabling the determination of the time intervals and frequency bands in which the induced amplitude modulation of brain electrophysiological oscillatory activity occurs.

From each dog, 122 ± 13 (mean ± SEM) single trials were included in the TFR analysis; data sequences included for the analysis had an impedance of approximately 8 ± 3 kΩ (across-dogs mean ± SEM). In the processing, the first trial in each block and trials in which the amplitude of any EEG channel exceeded 200 µV, indicating muscle activity or eye movements, were excluded. In addition, ICA was applied, similarly as for the analysis spontaneous rhythmic activity, to further reduce the effects of muscle artefacts. The time-frequency power was computed for each single trial from 0 to 1000 ms of the stimulus onset and for the frequencies of 1 to 40 Hz (with 1-Hz frequency intervals), using complex Morlet wavelets [25], [27]. In humans, wavelet analysis has been utilized successfully in neurophysiological studies for evaluating modulation of rhythmic activity (e.g., [28]) and cortical interactions [29]. The wavelet width of 7 was applied, allowing the best compromise between temporal and frequency resolution (see e.g., [30]). The wavelet-convolutions were first calculated separately for each trial between the 40 different Morlet wavelets and an epoch interval of −700 to 1500 ms with regard to the stimulus onset. Thereafter, the obtained TFRs were averaged across the trials. The individual dog TFRs were normalized with respect to the maximum modulation, calculated as a largest difference between the TFR values during the 0–1000 ms period of the stimulus onset (STIMULUS) and the −200 to 0 ms BASELINE period of each dog. This was done in order to exclude any individual outlier driving the group-level TFR effect.

To compare the induced oscillatory activity during the visual STIMULUS to the frequency content during the BASELINE period, the grand average TFRs and statistical maps were calculated for 0–1000 ms from the stimulus onset, with intervals of 50 ms and a time windows of 100 ms. This resulted in 21 × 40 partially overlapping time windows (with the first window at −50 to 50 ms representing the frequency content at time zero and the last window at 950 to 1050 ms representing the frequency content at time 1000 ms). In the group level statistical testing, the power of each of these STIMULUS windows was compared to the BASELINE power at the same frequency with paired-samples t-tests. Time-frequency clusters containing at least 3 adjacent time-frequency bins with *P*<0.001 were deemed to represent significant modulation of activity.

## Results

### Modulation of the 15–30 Hz spontaneous oscillatory activity by visual task

**Figure 3:**
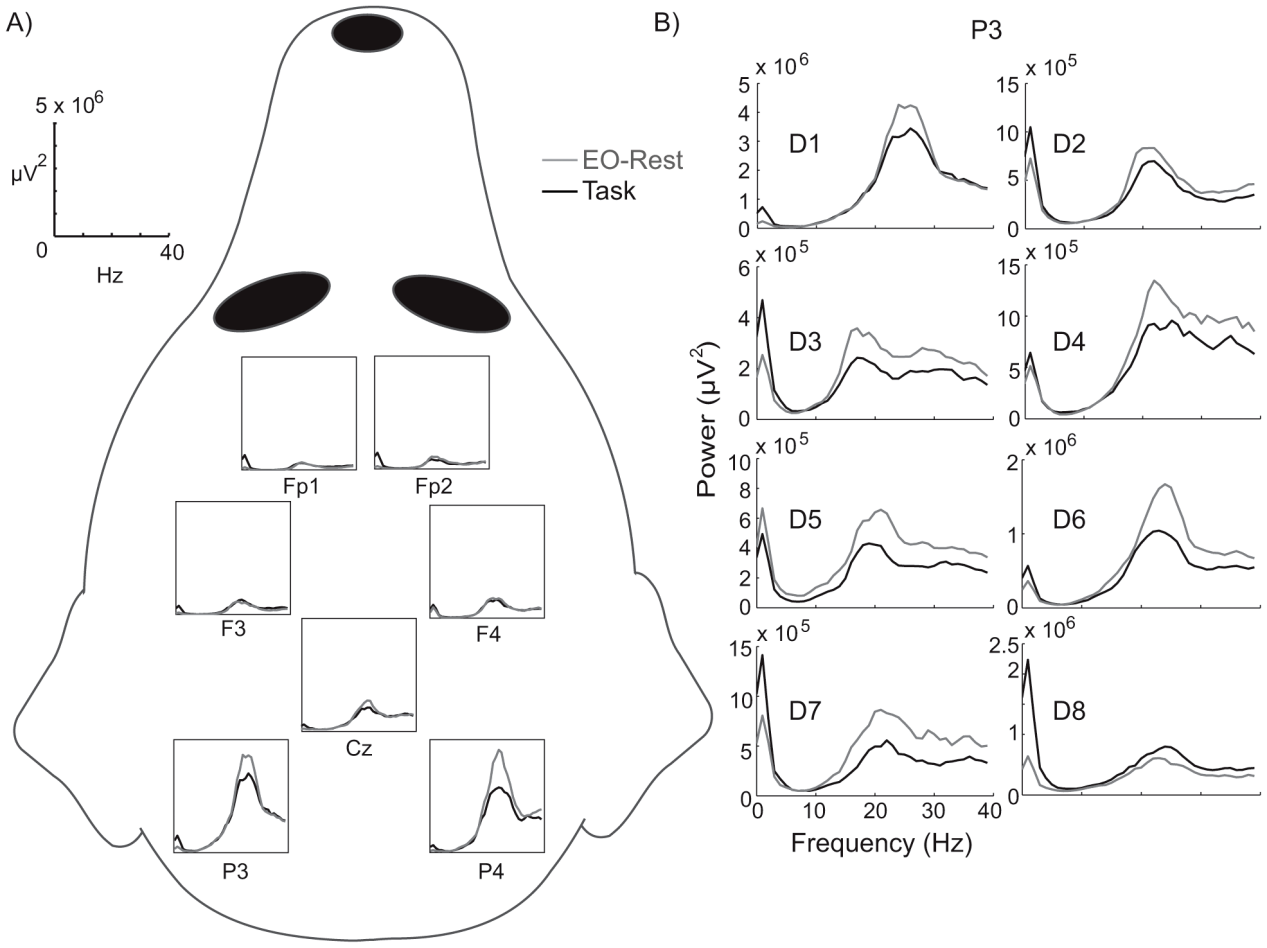
Frequency spectra during the stimulus block and at rest. A) An example from one dog illustrating the layout of the EEG channels as viewed directly from above; the units are given at the top left. B) The spectra of all dogs from the channel P3 at 0–40 Hz; the power has been scaled individually for each dog. Gray  =  EO-Rest; Black  =  Task.

The analysis of power spectra revealed a modulation of the spontaneous oscillatory activity related to the ongoing visual task. The oscillatory activity at the frequency band of 15 to 30 Hz peaked at the most posterior (occipital) sensors of P3 (mean peak frequency 23 Hz) and P4 (mean peak frequency 24 Hz) in all eight dogs, and it was suppressed during TASK compared to the EO-REST in 7 out of 8 dogs (see Figure 3A for an example from one dog). In 5/8 dogs, the 15–30 Hz activity was bilaterally detected in P3 and P4 sensors, and in 3/8 dogs the activity was slightly lateralized to the P3 sensor over the left hemisphere (Figure 3B).

At a group level, the task-related oscillatory activity was suppressed statistically significantly as compared with resting activity within the channel P3, at the 23 ± 3 Hz window around the peak frequency (*P*<0.01, T = 3.85, *df* = 7; paired-samples t-test). Within the channel P4, the suppression did not reach statistical significance (*P* = 0.34, T = 1.03, *df* = 7).

### Event-related suppression of the 2–6 Hz induced oscillatory activity

**Figure 4:**
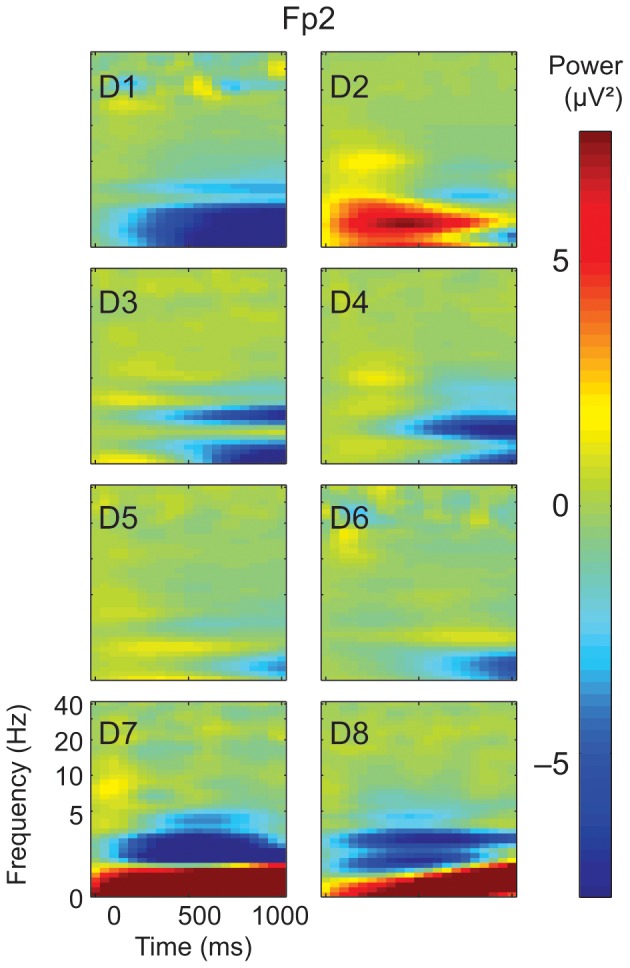
Suppression of the induced oscillatory activity. Modulation of the oscillatory activity (0–1000 ms from the onset of the stimulus presentation) in channel Fp2 in all individual dogs D1–D8. The modulation is shown as time-frequency representations within a logarithmic frequency scale. Color bar at right represents the power values.

At the group-level, the TFRs revealed a significant suppression of the induced oscillatory activity at the frequencies of 3–5 Hz during the visual STIMULUS, compared with the BASELINE 600–900 ms after stimulus onset; this effect was evident bilaterally in the most frontal locations of the sensor layout (in the channel Fp1: *P*<0.001, cluster-level T = −6.30, *df* = 7 and in the channel Fp2: *P*<0.001, cluster-level T = −5.72, *df* = 7; paired-samples t-test; at least 3 adjacent time-frequency bins). At the Fp1 and Fp2 sensors, this suppression was detected at the frequencies of 2–6 Hz in all individual dogs (TFRs of channel Fp2 shown in Figure 4); in addition, the effect was observable also at the F3 and F4 sensors in 7/8 dogs.

## Discussion

### Non-invasive visual EEG of domestic dogs

The neural signals obtained with the EEG were first observed in intracranial recordings from animals—rabbits and monkeys—by Caton [31], and after over hundred years of its employment in cognitive neuroscience in healthy humans, non-invasive measurement of EEG from the top of the skin is now possible also in animals [32], [33]. Our current data confirm that, via extensive behavioral training with positive reinforcement, it is possible to conduct non-invasive EEG measurement and group-level studies with fully conscious, non-medicated and unrestrained domestic dogs—an endeavor that has been deemed unsuccessful in the past [14], [34]. In our study, the training and the EEG recordings were optimized for the relatively short attention span, as well as vibrant habitude of dogs, compared with the standard measurements in human subjects. Furthermore, the stimuli of the experiment were ecologically valid faces, which have been previously found effective in non-invasive neurophysiological visual experiments in humans (e.g., [35]–[38]) and in an eye tracking experiment in dogs [8].

The training period needed for this experiment was relatively long compared to either human EEG or animal behavioral studies. Staying still for a long period of time without sleeping is rather challenging for many species, thus the training times needed for animal brain research are usually arduous and commonly, only one or two animals are trained for the task. In a recent experiment, scalp-EEG was measured from one chimpanzee, for whom the training took 0.5 years and the recording 50 days [33]; this one individual already trained for the task was also the subject for the subsequent experiments [39], [40]. In intracranial EEG experiments of macaque monkeys, the animal training time is often not mentioned, or noted simply requiring “lengthy” training (e.g., [41]–[43]). In a recent dog fMRI experiment, two dogs were taught for the brain scanning for 2 months [44]; however, the 2 dogs were already pre-selected on the basis of their curiosity and quick learning skills, whereas the 8 dogs of this study did not live among humans and were not accustomed to be separated from their group or trained for behavioral tasks, which partly explains the difference in the training times. Dogs were individually habituated to the testing environment and accustomed to the task gradually by an experienced animal trainer, to avoid any stress caused by new situations. Furthermore, our sample were not taught full-time but only twice a week for a short period of time, thus the training time needed might be diminished by full-time training and in cooperative family dogs. The same training procedure has taken less time with pet dogs, when only eye tracking was measured [8].

Today, in standard human non-invasive neurophysiological measurements, around 600 visual stimuli can be shown to the subjects during one measurement session lasting from 0.5 to 1.5 hours. In our study, the stimulus procedure was optimized through testing, and the stimulus rate of 8–12 consecutive stimuli before a break with a reward was found optimal for keeping the attention of the dogs. Furthermore, the average measurement time of 20 minutes per day was found as an optimal trade-off between the amount of stimuli and the attention of the dog.

### Spontaneous visual oscillatory activity of dogs

Our first major finding was the suppression of the spontaneous oscillatory activity at the frequencies of 15–30 Hz (so called beta range) during the visual TASK compared to the EO-REST in the most posterior channels: the phenomenon was robust in all but one dog. In our previous experiment, the most posterior channels P3 and P4 have shown the most prominent evoked visual N100 responses of dogs, strongly suggesting that these channels show the brain activity of the occipital cortex best out of our 7 sensors [45]. In the early, intracranial EEG studies of dogs, spontaneous brain rhythms roughly at the beta range (around 20–30 Hz) have been found from the occipital cortex during the dogs' awake state [15]. These first recordings found a predominant 20–30 Hz contribution, and noted a very small level of the spontaneous oscillatory alpha component (around 8–13 Hz) within the lateral and middle occipital cortex of awake dogs whose eyes were open [15], reminding our current non-invasive EEG recordings.

In humans, the spontaneous rhythmic activity at the beta range of the spectrum is connected to the sensorimotor activity and is most prominent in the somatomotor cortex, whereas visual processing in humans has been mostly associated with alpha-range activity (for review, see [18]). Task-engagement causes suppression of the spontaneous occipital alpha rhythm in humans (e.g., [46]–[51]). The subject engagement to a task is generally seen as a cognitive state linked to attention and concentration, and it is inversely related to the amount of cortical resources allocated to task performance [50]. The recordings of the alpha rhythm are best conducted with eyes closed, leading to more prominent power levels [18]; however in humans, the alpha rhythm is generally also detectable during rest with eyes open, without additional visual input except for the measurement environment and a blank screen (see e.g., [52]). Furthermore, the alpha rhythm is further suppressed in humans during visual stimulation (attended pictures vs. fixation without visual stimuli, see e.g., [53]), suggesting a strong reactivity of the alpha band in humans even with eyes open.

Intracranial studies measuring the neuronal activity dogs directly from the brain tissue have shown the appearance of the alpha-range activity when the dog closes its eyes, and its disappearance when the dog opens its eyes [54], [55]. After the period of dog resting with eyes closed, Lopes da Silva and colleagues [15] state: “At the end of such a period the dog either opened his eyes, and the alpha rhythm was immediately replaced by activity at higher frequencies” (p. 628). With our current non-invasive EEG measurements, we found a strong contribution of the beta rhythm when the dogs' eyes were open, in line with the earlier literature [15], [54], [55]. Additionally, the beta rhythm was suppressed during the TASK with more visual stimulation (faces) compared to the EO-REST, showing modulation of the rhythm according to the ongoing visual stimulation.

Previous intracranial measurements have shown the visual, attentive processing affecting the beta-range activity also in cats [56]–[61]. Some of studies have reported beta activity within the posterior parietal area, during motionless visual fixation of the cat [57]–[59]. Although the behavioral setting reminds our experiment with dogs, the frequency contributions in those studies seem to be somewhat higher (around 35–45 Hz) than those found in our current study (15–30 Hz, peaking around 20 Hz). Another set of studies have found 20 Hz oscillatory peaks during visual attention from the primary visual cortex of the cat [56], [60], [61]. Although with non-invasive EEG alone, the absolute origin of the detected signals cannot be confirmed, the latter set of studies remind our findings in both frequency and more posterior spatial location. The posterior location of the channels, together with our previous data with the most prominent visual N100 responses within these channels [45], also suggest that these channels show the activity best from the visual/occipital cortices of dogs. Nevertheless, we cannot rule out the possibility that part of the beta-range activity detected here may be generated within the parietal cortices of the dogs.

### Induced oscillatory suppression during visual stimulus

The second major finding of the current study was the suppression of the induced oscillatory activity at the frequencies around 2–6 Hz during the visual stimulus, as revealed by the TFRs especially at the most frontal sensors. This suppression was strictly time-locked to the visual stimulus onset, as it was not detected at the more global spectral analysis of the data that included also the inter-stimulus intervals between stimuli and showed more larger-scale modulations within the data.

Analogous time-locked suppression of an oscillatory rhythm is present within the mu-rhythm, comprising 10 Hz and 20 Hz components, in the human somatomotor cortex during movement (for reviews, see [18], [22]), and similar motor suppression has also been found in monkeys [62], [63] and in cats [58]. The mu-rhythm of humans is a prominent ongoing background rhythm during rest, but suppressed during any kind of movement, even as small movement as finger tapping [64]. In humans, the level of the motor rhythm starts to suppress about 2–1.5 seconds before a voluntary finger movement, it recovers to the baseline level in 0.5–1 seconds, and is followed by a 1–2 second rebound, a period of activity stronger than the baseline.

In our study, the dogs were free to explore the stimuli with eye fixations, thus each visual stimulus initiated a movement of the dogs' eyes; the signal caused by the movement of the eyes themselves is captured by the spectral analysis in Figure 3 (higher 1 Hz peak during the visual task than at rest). Accordingly, it is possible that the suppression of the ca 2–6 Hz frontal activity, present in all individual dogs during the stimulus presentation, reflects a motor rhythm related to the exploratory eye movements. The source of the rhythm might be either directly in the motor cortex, or within the homologue of frontal eye fields (FEF) in dogs, since both of these are more frontal in the dog than in the human brain and are likely to be captured by our frontal sensors.

Although the stimulus images in this study consisted of face images of dogs and humans, they were used only due to their ecological relevance for the dogs and due to the parallel eye tracking experiment with a different agenda. The possible category-related differences were not the target of this study, thus, the stimulus images were not rendered fully comparable (e.g., different frequencies of stimulus occurrences) and the current data set does not quantify the possible differences between different types of stimuli. However, the current methodological setup enables the possible comparison across stimulus categories in the future.

### Response variability among dogs and the across-species comparison

Our current results show a remarkable variance among individual dogs, both in the induced and sustained brain oscillatory activity. Also in human measurements, large variability is observed in oscillatory activity (e.g., in [52], [65]), most likely reflecting both physiological and methodological differences during measurement conditions; the current variability in dogs likely reflects similar processes. The impedance of the electrodes varies across dogs and across measurement days; the artefact-free EEG samples vary also accordingly, both of which can affect the resulting signal-to-noise ratio and individual results. Also cognitive events, the dogs' vigilance and attention to the task, as well as subtle differences in the brain structure may affect the data.

The comparison of the current data set to the previous studies on dog visual cognition is challenged by the differences in the methodologies used. The previous intracranial EEG measurements on dogs have required anesthesia of the animal, causing relaxed drowsiness, whereas in our study, the dogs were extremely vigilant and alert and only staying still for short periods of time due to positive operant conditional reinforcement. Furthermore, the earlier intracranial measurements have enabled the data collection from the different cortical layers, whereas the non-invasive EEG detects signals that are strong enough to be detected at the skin.

Methodologically, more similar studies have been conducted in humans. However, direct comparisons across species are not straightforward as the human brain is likely to generate stronger currents at the top of the skin due to its larger volume and smaller distance to the skin. Moreover, the evolutionary distance may also cause differences in the functionality of the oscillatory frequencies between species. Evolutionarily differentiation of the network properties of neurophysiological oscillations are not completely resolved within previous literature: however, our work suggests some differences in the basic network functionality between dogs and humans, worth more detailed attention in the future.

## Conclusions

We demonstrate the measurement of the brain activation of domestic dogs in a completely non-invasive fashion, based on intensive operant-positive reinforcement training. Our study shows, to our knowledge, the first group-level data of dog visual perception, and our results point to both similarities and differences within the basic functionality of the dog compared to human cognitive neurophysiology. At this stage, the current data set relates more closely to the fundamental aspects of perceptual experience across species rather than to the behavioral experiments with dogs. However, our results demonstrate the feasibility of non-invasive EEG oscillatory recordings, measured with adhesive electrodes attached to the top of the skin, in dog visual cognition. Thus, the study opens the possibility to implement cognitive neuroscience studies with dogs and to examine the evolutionary background and divergence of brain function associated with cognition.
